# Comparative analyses of 32 complete plastomes of Tef (*Eragrostis tef* ) accessions from Ethiopia: phylogenetic relationships and mutational hotspots

**DOI:** 10.7717/peerj.9314

**Published:** 2020-06-19

**Authors:** Girma Eshetu Teshome, Yeshitila Mekbib, Guangwan Hu, Zhi-Zhong Li, Jinming Chen

**Affiliations:** 1 CAS Key Laboratory of Aquatic Botany and Watershed Ecology, Wuhan Botanical Garden, Chinese Academy of Sciences, Wuhan, Hubei, China; 2 Center of Conservation Biology, Core Botanical Gardens, Chinese Academy of Sciences, Wuhan, Hubei, China; 3 Sino-Africa Joint Research Center, Chinese Academy of Sciences, Wuhan, Hubei, China; 4 University of Chinese Academy of Sciences, Beijing, China

**Keywords:** *Eragrostis tef*, Plastome, Molecular barcoding, Polymorphic regions, Phylogenetic analysis

## Abstract

*Eragrostis tef* is an important cereal crop in Ethiopia with excellent storage properties, high–quality food, and the unique ability to thrive in extreme environmental conditions. However, the application of advanced molecular tools for breeding and conservation of these species is extremely limited. Therefore, developing chloroplast genome resources and high-resolution molecular markers are valuable to *E. tef* population and biogeographic studies. In the current study, we assembled and compared the complete plastomes of 32 *E. tef* accessions. The size of the plastomes ranged from 134,349 to 134,437 bp with similar GC content (∼38.3%). Genomes annotations revealed 112 individual genes, including 77 protein-coding, 31 tRNA, and 4 rRNA genes. Comparison of *E. tef* plastomes revealed a low degree of intraspecific sequence variations and no structural differentiations. Furthermore, we found 34 polymorphic sites (13 cpSSRs, 12 InDels, and 9 SNPs) that can be used as valuable DNA barcodes. Among them, the majority (88%) of the polymorphic sites were identified in the noncoding genomic regions. Nonsynonymous (ka) and synonymous (ks) substitution analysis showed that all PCGs were under purifying selection (ka/ks <1). The phylogenetic analyses of the whole plastomes and polymorphic region sequences were able to distinguish the accession from the southern population, indicating its potential to be used as a super-barcode. In conclusion, the newly generated plastomes and polymorphic markers developed here could be a useful genomic resource in molecular breeding, population genetics and the biogeographical study of *E. tef*.

## Introduction

The genus *Eragrostis* comprises approximately 400 morphologically distinct species distributed throughout the subtropical and tropical regions of the world (Clayton et al., 2016). *Eragrostis tef * (Zucc.) Trotter is the sole species in the genus *Eragrostis* cultivated for human consumption and Ethiopia is the center of origin and genetic diversity for *E. tef* (Ketema, 1997). Compared to other cereal crops, *E. tef* is more tolerant of extreme environmental conditions and is therefore considered as lower risk crop (Assefa et al., 2015). These characteristics, together with its grain nutrition, market value, desirable storage properties, make this crop attractive to smallholder farmers (Minten, Taffesse & Brown, 2018). The grain of *E. tef* is also gaining global popularity as healthy and high-performance food due to its high fiber contents and gluten-free nature (Spaenij-Dekking, 2005; Chanyalew et al., 2019). The long history of cultivation and variety selection coupled with the broad agro-ecology adaptation of the crop resulted in high genetic diversity in Ethiopia (Assefa, Chanyalew & Tadele, 2017). Currently, more than 5,000 *E. tef* accessions collected from different geographic regions of Ethiopia are preserved* * in the seed gene bank of the Ethiopia Biodiversity Institute (EBI; Tesema, 2013). The conserved accessions are the main sources of genetic variations to enrich the genetic base of cultivated varieties. To establish proper conservation and efficient utilization of the plant genetic resource, understanding genetic variations between and within gene bank samples is essential (Wambugu, Ndjiondjop & Henry, 2018). However, the studies of genetic diversity among accessions of *E. tef* are still highly limited and one of the most important reasons is the lack of effective molecular markers (Tadele, 2018; Chanyalew et al., 2019).

Advances in biotechnology, especially in the area of molecular biology has provided some critical tools for proper conservation and use of plant genetic resources (Yuan et al., 2017). From the perspective of improving crops through modern breeding programs, molecular markers have played significant roles, especially in the determination of genetic diversity and the classification of germplasm (Majeed et al., 2015; Nadeem et al., 2017). During the last few decades, several universal molecular markers such as amplified fragment length polymorphism (AFLP) (Bai et al., 1999), simple sequence repeats (SSR) (Abraha et al., 2016), random amplified polymorphic DNA (RAPD) (Bai et al., 2000) and inter simple sequence repeat (ISSR) (Assefa, Merker & Tefera, 2004) have been used in *E. tef*. Moreover, first draft genome (Cannorazzi et al., 2014) and chromosome-scale genome assembly (VanBure et al., 2020) of *E. tef* have been made publicly available online. These molecular studies have provided some insight into *E. tef* population genomics and phylogenetic relationships. Furthermore, the utilization of some universal chloroplast markers for the phylogenetic studies have been reported in previous studies (Espelund et al., 2000; Ingram & Doyle, 2003). The available reports concerning the *E. tef* plastome sequence variability are insufficient for population genetics and biogeographic studies (Assefa et al., 2011; Assefa et al., 2015. Also, there is no valuable molecular barcoding system to discriminate and classify the conserved accessions according to their geographical regions of collection. These will have a direct effect on the conservation and the sustainable utilization of the crop. Therefore, sequencing and comparative analysis of the plastome have the potential to detect intraspecific polymorphism and provide useful molecular markers for various studies in *E. tef*.

The plastome is commonly characterized by an extremely conserved structure and possessed a relatively slow evolutionary tempo (Greiner, Sobanski & Bock, 2015). It generally comprises a pair of inverted repeats (IRs) regions, one large single-copy (LSC) region and one small single-copy (SSC) region (Brears, Schardl & Lonsdale, 1986). Although overall plastome structure is always thought to be conserved, structural variations such as inversion (Lei et al., 2016; Kim & Cullis, 2017), gene duplication and IR boundary shifts (Zhu et al., 2016) have been detected among angiosperms. For the mutations of sequences, the single nucleotide polymorphism (SNP) and deletion or insertion (InDels) of nucleotide bases are the most common variations in the sequences of plastome (Decesare, Hodkinson & Barth, 2010; Kim et al., 2015). These variations have provided ideal information for developing polymorphic markers for numerous applications such as molecular barcoding (Okoth et al., 2016; Zong et al., 2019), phylogenetic reconstruction (Peterson, Romaschenko & Johnson, 2010), biogeographic studies (Kress et al., 2009; Xie et al., 2019), assessment of the maternal line of hybrid species (Schroeder, Höltken & Fladung, 2011; Tomar et al., 2014; Chung et al., 2019), and clarification of the evolutionary relationship between cultivated and crop wild relatives (Gao et al., 2019). Currently, with the advancement of next-generation sequencing technologies (NGS), sequencing of chloroplast genomes and the development of plastid genetic markers have become feasible in various plant genetic researches (Brozynska, Furtado & Henry, 2014; Bi et al., 2018).

In this study, the complete plastomes of 32 *E. tef* accessions were newly sequenced and assembled. All these plastomes were compared to examine the intraspecific chloroplast genomes sequence variability, to our knowledge, to gain the first compressive analysis of plastome structural variations and mutations across *E. tef* plastome. Specifically, the distribution of chloroplast simple sequence repeats (cpSSRs), single nucleotide polymorphism (SNPs) and InDels regions were investigated. Besides, phylogenetic analysis was performed to evaluate the genetic relationship of the studied accessions with their respective biogeographic distribution using variable sites detected in the present study. We also examined the impact of identified sequence variations on the evolution of protein-coding genes (PCGs). The markers could be a useful genomic resource for use in various studies such as molecular breeding, molecular barcoding, biogeography and population genetic diversity studies in *E. tef*.

## Material and Methods

### Plant sampling and DNA extraction

**Figure 1:**
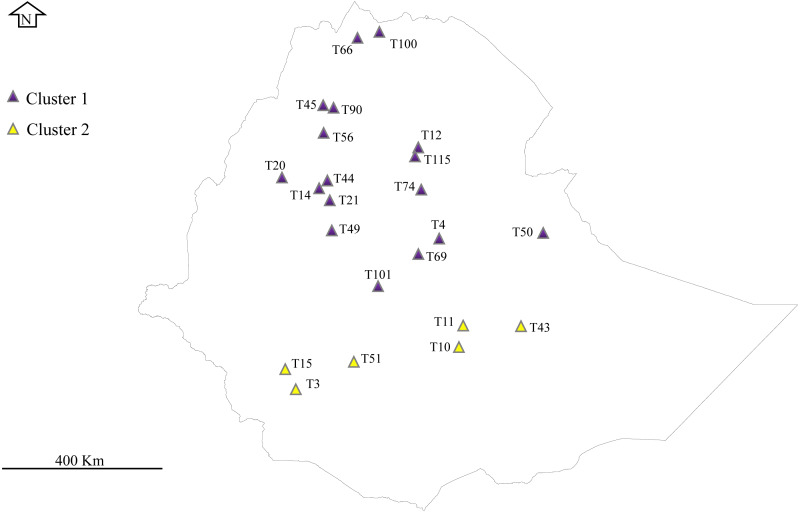
Phylogeographical distribution of sampled * E*. *tef* accessions. The accessions collected from southern Ethiopia (cluster 2) were represented by yellow color. The blue color represents the accessions originated from the north and central part of Ethiopia. Sample without detail GPS points, including T1, T16, T24, T34, T36, T68, T93, T81 and T116 were not represented in the map. DIV-GIS software was used to show the GPS location of the accessions collection sites in Ethiopia.

A total of 32 *E. tef* accessions were obtained from the Ethiopian Biodiversity Institute (EBI) seed genebank. These accessions were sampled from Amhara, Benishanguz Gumuz, Tigray, Oromia, and Southern regions, representing the geographic distribution of the species in Ethiopia (Fig. 1, Table 1). Ethiopian Biodiversity Institute approved this study (EBI 712222942018). The collected leaves were dried immediately using silica gel and preserved in the refrigerator (−20 °C) until DNA extraction. Total genomic DNA was isolated from the dried leaf of each accession using the MagicMag Genomic DNA Micro Kit (Sangon Biotech Co., Shanghai, China) following the protocol given by the manufacturer. The purity and quality of the DNA were detected by electrophoresis on the 1% agarose gel.

### Chloroplast genome sequencing, assembly and annotation

Short inserts of ∼350 bp DNA sequencing library for each sample was constructed using TruSeq DNA sample preparation kits (Illumina, San Diego, CA, USA). And 150 bp paired-end reads sequencing was carried out using the Illumina Hiseq 2500 Platform (Illumina, San Diego, CA) at the Beijing Genomics Institute (Shenzhen, China). Approximately 10G raw data of each sample was generated, then filtered using Fastp with default parameters (Chen et al., 2018). The remaining clean reads were de novo assembled using NOVOPlasty 2.7.1 (Dierckxsens, Mardulyn & Smits, 2017) with Kmer 31–39, where *E. tef* (Gene bank accession no. NC_029413) was used as the seed and reference sequence. Finally, only one contig per accession was generated, then we remapped them against the previously published plastome of *E. tef* (NC_029413) using the software GENEIOUS R 8.0.2 (Kearse et al., 2012). Annotation of the assembled genomes was performed using the GeSeq (https://chlorobox.mpimp-golm.mpg.de/geseq.html; Tillich et al., 2017). In order to confirm the accuracy of annotation, each annotated gene was checked for start and stop codons using the software GENEIOUS R 8.0.2 (Kearse et al., 2012) manually. A circular map for the plastome was drawn using the OrganellerGenomeDraw 1.3.1 (OGDRAW) (Greiner, Lehwark & Bock, 2019). For the structural comparison, alignments of 32 plastomes were compared using mVISTA software (Frazer et al., 2004). In order to detect the IR expansions/contraction, all the annotated plastome sequences for the 32 *E. tef* accessions were compared to the LSC, SSC and IRs border using an online program IRscope (https://irscope.shinyapps.io/irapp/; Amiryousefi et al., 2018). All annotated plastome sequences were submitted to the National Center for Biotechnology Information (NCBI) database (accession numbers: MN780987 to MN781018).

**Table 1:** The feature of 32 *E. tef* plastomes and geographic information of accessions.

No	Sequence code	Original country	Latitude	Longitude	Altitude (m)	Genome size	LSC (bp)	IR(bp)	SSC(bp)	EBI voucher	Genebank accession ID
1	T1	ETH	11°28′00″N	39°17′00″E	1,900	134,350	79,726	21,022	12,580	234,760	MN781003
2	T3	ETH	05°59′00″N	37°32′00″E	1,250	134,437	79,795	21,021	12,600	235,659	MN781007
3	T4	ETH	08°50′93″N	39°00′00″E	NA	134,351	79,728	21,021	12,581	221,627	MN781018
4	T10	ETH	06°58′49″N	40°29′11″E	1,783	134,349	79,726	21,021	12,581	28658	MN781011
5	T11	ETH	07°01′23″N	40°20′56″E	2,140	134,352	79,729	21,021	12,581	28660	MN781004
6	T12	ETH	11°23′00″N	39°19′00″E	2,630	134,352	79,729	21,021	12,581	234,764	MN780995
7	T14	ETH	10°27′00″N	37°02′00″E	2,440	134,349	79,726	21,021	12,581	55172	MN781009
8	T15	ETH	06°02′32″N	37°24′57″E	2,338	134,418	79,794	21,021	12,582	29751	MN781006
9	T16	ERT	NA	NA	NA	134,358	79,733	21,022	12,581	233,294	MN781010
10	T20	ETH	10°59′00″N	36°38′00″E	1,815	134,351	79,728	21,021	12,581	243,553	MN780992
11	T21	ETH	10°02′38″N	37°22′15″E	2,048	134,350	79,727	21,021	12,581	26358	MN781013
12	T24	ETH	NA	NA	1,600	134,421	79,797	21,021	12,582	202,439	MN780993
13	T34	ETH	NA	NA	2,800	134,351	79,728	21,021	12,581	206,841	MN780989
14	T36	ETH	NA	NA	1,550	134,350	79,727	21,021	12,581	236,495	MN780991
15	T43	ETH	07°03′53″N	41°04′00″E	1,248	134,419	79,795	21,021	12,582	28561	MN780994
16	T44	ETH	10°41′00″N	37°22′00″E	1,890	134,349	79,726	21,021	12,581	234,720	MN781012
17	T45	ETH	12°21′00″N	37°31′00″E	1,920	134,351	79,728	21,021	12,581	243,537	MN781005
18	T49	ETH	09°26′00″N	37°07′00″E	2,340	134,349	79,726	21,021	12,581	55263	MN780999
19	T50	ETH	09°30′00″N	42°37′00″E	1,925	134,351	79,727	21,022	12,582	29754	MN781017
20	T51	ETH	06°30′57″N	38°34′14″E	2,563	134,422	79,798	21,021	12,582	55126	MN780996
21	T56	ETH	11°45′00″N	37°05′00″E	1,955	134,351	79,728	21,021	12,581	242,143	MN780990
22	T66	ETH	14°06′00″N	38°09′00″E	1,300	134,350	79,727	21,021	12,581	238,202	MN780987
23	T68	ETH	NA	NA	NA	134,349	79,726	21,021	12,581	236,738	MN781015
24	T69	ETH	08°50′00″N	39°20′00″E	1,700	134,351	79,728	21,021	12,581	236,957	MN781002
25	T74	ETH	10°32′00″N	39°55′00″E	1,480	134,349	79,726	21,021	12,581	237,133	MN781000
26	T81	ETH	NA	NA	2,144	134,412	79,789	21,021	12,581	244,855	MN780988
27	T90	ETH	12°17′00″N	37°44′00″E	1,855	134,350	79,727	21,021	12,581	242,187	MN781001
28	T93	ETH	NA	NA	2,320	134,355	79,732	21,021	12,581	236,525	MN780997
29	T100	ETH	14°12′00″N	38°56′00″E	2,020	134,350	79,727	21,021	12,581	237,210	MN780998
30	T101	ETH	07°50′00″N	39°05′00″E	1,740	134,350	79,727	21,021	12,581	237,578	MN781016
31	T115	ETH	11°08′00″N	39°13′00″E	3,090	134,414	79,790	21,021	12,582	243,491	MN781008
32	T116	ETH	11°08′00″N	39°13′00″E	3,090	134,350	79,727	21,021	12,581	243,503	MN781014

**Notes.**

ETH Ethiopia ERT Eritrea EBI Ethiopian Biodiversity Institute seed bank accession number NA not available

### Screening variable regions and intraspecific comparison

Considering the wide range of cpSSR markers applications in the breeding scheme, population and phylogenetic studies (Melotto-Passarin et al., 2011; Diekmann, Hodkinson & Barth, 2012; Ebrahimi et al., 2019), Firstly, we detected the location and types of cpSSRs in the plastome of *E. tef* accessions using MISA perl script (Beier et al., 2017). The minimum number of repeat unit was adjusted to eight, six, five, five, three, and three, for mononucleotides, dinucleotides, trinucleotides, tetranucleotides, pentanucleotides, and hexanucleotides, respectively. We then employed REPuter (Kurtz et al., 2001) to identify four types of large repeating sequences (reverse, forward, complement and palindromic) with a minimum repeat size of 30 bp, hamming distance equal to 3 and maximum computed repeats was set to 50 bp. To compare the cpSSR of *E. tef* with related species, three chloroplast genomes were chosen from sub-family Chlorodoideae including *Eragrostis minor* (NC_029413), *Neyraudia reynadiana* (NC_024262)*,* and *Melanocenchris abyssinica* (NC_036694)*and cpSSRs were detected using MISA (Beier et al., 2017))* with same settings. Multiple alignments of 32 plastomes performed using an online program MAFFT 7 (Katoh, Rozewicki & Yamada, 2017) with default parameters, and then mapped to reference genome using GENEIOUS R 8.0.2 (Kearse et al., 2012). Using the cpSSR information of T3 as the reference, we screened the variable cpSSR among the aligned plastomes of all accessions. After masked the polymorphic cpSSR regions, we further identified the SNPs and InDels separately, as well as their positions in the mapped genome. Additionally, if the polymorphic positions located in the coding sequences, we aligned the sequences using GENEIOUS R 8.0.2 (Kearse et al., 2012) to analyze further if there are any changes in the amino acid of the gene containing variable sites. The primers for all identified variable regions were designed using the online Primer 3.0 (http://bioinfo.ut.ee/primer3/) program with default parameters.

### Phylogenetic analysis

The phylogenetic trees were constructed using two data sets: (1) the complete plastome sequences of 32 *E. tef* accessions (2) concatenation of sequences extracted from twenty polymorphic regions (SNPs and InDels) identified in the current study. Sequence length was determine based on the designed PCR product and was tested for their performance in delineating accessions based on their phylogeographic origin. Before the phylogenetic tree construction, one copy of the IR was removed from the complete chloroplast genome. All sequences alignment was accomplished using MAFFT 7 (Katoh, Rozewicki & Yamada, 2017) plugin in Phylosuite 1.2.1 (Zhang et al., 2019). The phylogenetic analyses were performed using maximum likelihood (ML) and Bayesian inference (BI). ModelFinder (Kalyaanamoorthy et al., 2017) was used to select the best-fit model with default setting and the maximum likelihood (ML) analysis was performed using IQ-TREE 1.6.12 (Trifinopoulos et al., 2016) with 1000 bootstrap replications. The BI analysis was performed by MrBayes 3.2.6 (Ronquist et al., 2012), with a total of 2,000,000 generations set to perform the analysis. Four chains run with sampling after every 3000 generations and the first 25% trees were discarded as burn-in, and the remaining trees were constructed a majority rule consensus tree.

### Analyses of signatures of selection

To detect the evidence of selective acting in mutational PCGs, the ratio of nonsynonymous (ka) to synonymous (ks) substitution (ka/ks) of mutational PCGs were calculated using DnaSP version. (Librado & Rozas, 2009). Each extracted PCGs with mutational was aligned using GENEIOUS R 8.0.2 (Kearse et al., 2012) and average pairwise values of ka/ks ratio were determined for all accessions.

## Results

### Feature of sequenced *E. tef* plastomes

The size of the complete plastome sequences of *E. tef* ranged from 134,349 to 134,437 bp (Table 1). They possess a pair of IRs regions (42,042–42,044 bp), one pair of IRs regions (42,042–42,044 bp), one LSC region (79,726–79,798 bp) and one SSC region (12,581–12,600 bp). The guanine-cytosine (GC) content of plastomes was approximately 38.3% and the IR region was slightly higher (44%) compared to LSC (36.3%) and SSC (32.1%) regions. For analysis of the IR junction (contraction/expansion), we compared the border between LSC/IRb/SSC/IRa of all 32 *E. tef* accessions, and also observed highly conserved IR junction sites (Fig. S1).

**Figure 2:**
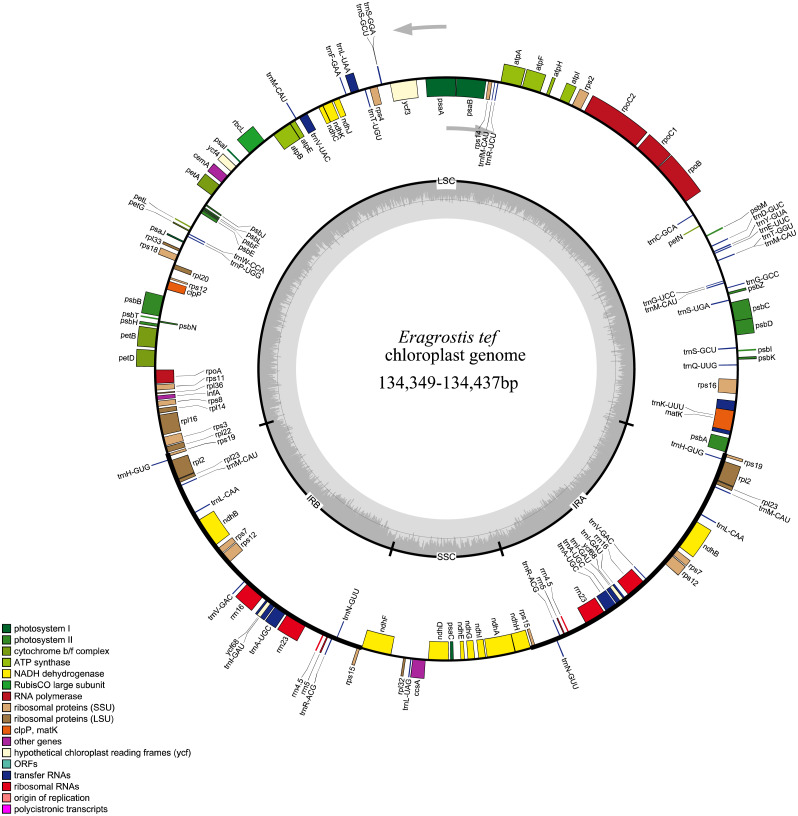
Whole plastome map of *E. tef*. Genes shown on the outside of the large circle are transcribed clockwise, while genes shown on the inside are transcribed counterclockwise.The thick lines indicate the extent of the inverted repeats (IRa and IRb), which separate the genome into small and large single-copy regions.

All 32 *E. tef* plastomes possessed common gene contents, which included a total of 112 individual genes, including 77 PCGs, 31tRNAs and 4 ribosomal RNA genes (Fig. 2). Among these, the LSC region contains 59 PCGs and 22 of them are tRNA genes, while 10 PCG and one tRNA genes are located in the SSC region. Eight PCGs (*rps7, rps12, rps15, rpl2 , rpl23 rps19, ndhB, yf68*), eight tRNA (*trnI-CAU, trnH-GUG, trnL-CAA, trnI-GAU, trnV-GAC, trnR-ACG, trnA-UGC, trnN-GUU*) and four rRNA genes (*rrn4.5, rrn5, rrn16, rrn23*) were duplicated in IR regions. Fifteen genes contained introns, of which nine of them are PCGs (*ndhA, ndhB, petB, petD, atpF, rps12, rps16, rpl2* and *rpl16*) and five tRNA genes (*trnA-UGC, trnV-UAC, trnK-UUU*, *trnG-UCC*, and *trnI-GAU*) had one intron, and *ycf3* gene contained three introns (Table S1).

### Simple sequence repeats

A total of 143 cpSSRs loci were identified in the plastome of *E. tef* accession (Table S2). The number of detected cpSSRs and their distributions are similar among compered accessions. These cpSSRs were mainly sited in the LSC region (78%), whereas 13% and 9% were localized in SSC and IR, respectively. The majority of cpSSRs were found in intergeneric space regions of the genome (73%) and the other 19% were located in the twelve PCGs (*rpoB*, *rpoC1*, *atpF*, *rps14*, *ndhK*, *ycf4*, *petA*, *petL*, *psaJ*, *psbB*, *rpl16*, *ndhF*, Table S2). The remaining 9% was located in the intron region. Among the cpSSR categories, mononucleotide cpSSRs are quite plentiful in the genome (94%), followed by dinucleotide cpSSRs (5%) and tetranucleotide cpSSRs (1%). No of tri-, penta- and hexa- repeat types were detected in the *E. tef*. The most common of a repeat mononucleotide was A/T (90%) motif. Thirteen cpSSRs sites are found polymorphic within *E. tef* accessions and all of them were situated in the LSC region of the genome (Table S3). Three plastomes were chosen from the subfamily *Chloridoideae* and their cpSSRs repeat number was compared with the *E. tef*. A total of 142, 141 and 118 cpSSRs were found in the *M. abyssinica*, *E. minor* and *N. reynaudiana,* respectively (Table S4). In addition to cpSSRs, large repeat sequences were analyzed using REPuter, and 44 repeats (Table S5), which include 28 forward (F), 15 palindromic (P) and one reverse (R) repeats, were found. There were no complement repeats in the *E. tef*. The repeat sequence that ranged between 30 to 40 bp were the most common (27 repeat loci). The majority (55%) of these repeats were located in the noncoding region of the plastome.

### SNPs and InDels polymorphism among *E. tef* accessions

After masked cpSSR regions, the intraspecific comparison of 32 *E. tef* accessions revealed 21 (12 InDels and 9 SNPs) polymorphic sites (Table 2). Of these, 16 sites were situated in the LSC region, and the SSC region only includes three sites. The IR regions contained only one variable site in *trnN-GUU-rps15*, which is one base deletion. The majority (81%) of the variable sites were located in the noncoding regions. Four of 21 variable sites were detected in PCGs (Table 2). Most of the SNPs were identified in the noncoding regions of the plastomes. T/C base substitutions accounted for the highest percentage (23%) of all SNPs, followed by T/A (15%), G/C (15%), G/T (15%), A/G (7.7%), G/A (7.75%), and A/T (7.7%). Besides, mutational sites identified in the PCGs (*atpE, psbB, ndhB* and *petB*) were classified as synonymous mutations (Table 2).

**Table 2:** Variable loci (SNPs, InDels) positions among 32 compared whole plastomes of *E. tef* accessions.

Location	Type	Region	Effect on protein	Synonymous (ks) value
*rps16 intron*	SNP	LSC		
*trnM-CAU-trnE-UUC* (*IGS*)	SNP	LSC		
*atpE* (*PCG*)	SNP	LSC	Synonymous	0.010
*clpP-psbB* (*IGS*)	SNP	LSC		
*psbB* (*PCG*)	SNP	LSC	Synonymous	0.002
*petB* (*PCG*)	SNP	LSC	Synonymous	0.006
*ndhB* (*PCG*)	SNP	IR	Synonymous	0.002
*rpl16 intron*	SNP	LSC		
*psaC-ndhE* (*IGS*)	SNP	IR		
*trnY-GAU-trnD-GUC* (*IGS*)	InDeLs	LSC		
*psaA-ycf3* (*IGS*)	InDeLs	LSC		
*petA-psbJ* (*IGS*)	InDeLs	LSC		
*trnT-UGU-trnS-UGA* (*IGS*)	InDeLs	LSC		
*ndhC-trnV-UAC* (*IGS*)	InDeLs	LSC		
*atpB-rbcL* (*IGS*)	InDeLs	LSC		
*rpl33-rps18* (*IGS*)	InDeLs	LSC		
*petD-rpoA* (*IGS*)	InDeLs	LSC		
*trnN-GUU-rps15 (IGS)*	InDeLs	IR		
*ccsA-ndhD* (*IGS*)	InDeLs	LSC		
*psaC-ndhE* (*IGS*)	InDeLs	SSC		
*psaJ-rpl33* (*IGS*)	InDeLs	LSC		

**Notes.**

PCG Protein coding gene IGS Intergenic spacer

The current study revealed that InDels were the abundant (12 InDels) type of polymorphism in the *E. tef* plastomes, and nearly all of them were found in the LSC region. Only one InDel was found in the SSC region (*psaC-ndhE*). The majority of InDels (81%) are single base pairs and all single base-pair InDels are A or T. Two InDels in the IGS regions (*trnY-GAU-trnD-GUC* and *psaA-ycf3*) gene were specific to the T16 accession. Thirty-four pairs of primer sequences (12 InDels, 9 SNPs, 13 cpSSRs) were developed based on the detected polymorphic sites in plastomes (Tables S3, S6).

### Phylogenetic analysis

**Figure 3:**
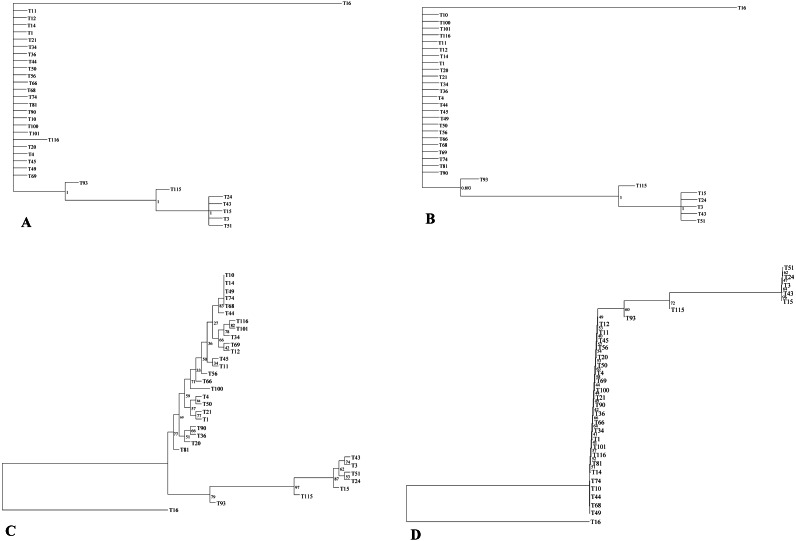
Phylogenetic relationships among *E. tef* accessions inferred from Bayesian (BI) (A, B) and maximum likelihood (ML) methods (C, D) using complete plastome sequences and twenty variable loci, respectively.

The phylogenetic relationship of 32 *E. tef* accessions was established using the complete plastome sequences and variable loci identified in the present study (Tables S3, S6). Both ML and BI gave identical tree topologies and clusters (Fig. 3). In the phylogenetic analysis, all *E. tef* accessions were divided into two clusters: one formed from accessions of south Ethiopia (Fig. 3) and others included the accessions from central and northern regions. Similarly, the phylogenetic tree inferred from twenty variable sites did show unambiguous biogeographic patterns in the accessions from the south (T3, T15, T24, T43, and T51) (Fig. 3). However, the phylogenetic relationships derived from both datasets did not provide clear biogeographic patterns.

### Selection analyses

We examined the pattern of nonsynonymous to synonymous substitution ratio (ka/ks) among four mutational PCGs (*atpE, psbB, ndhB* and *petB*) of *E. tef* accessions. The highest average ks pairwise value was found in *atpE* (0.010) (Table 2). The ka/ks ratio for PCGs showed zero values for all analyzed accessions.

## Discussion

### Plastome variations in *E. tef*

In this study, we conducted whole plastome comparison and determined the site of mutational changes in *E. tef*. The intra-specific comparison among 32 *E. tef* accessions revealed similar genome structure and no IR region expansion or contraction has occurred within the accessions. The result suggests that the *E. tef* plastome sequence is highly conserved (Figs. S1; S2). This finding was similar to other studies showing low intraspecific genetic variation (Jiang, Hinsinger & Strijk, 2016; Jeon & Kim, 2019). Although the plastomes composition and structures of 32 *E. tef* accessions are highly conserved, we also identified several mutational regions containing variable loci, which could provide potential information for the development of molecular marker and evolutionary studies. In our study, 143 cpSSRs identified in the *E. tef*, including thirteen polymorphic cpSSRs. The distributions of cpSSRs are non-random and a similar number of repeats among *E. tef* accessions. The number of cpSSRs detected in the *E. tef* was also relatively similar to other species in the subfamily *Chloridoideae* including *E. minor*, *M. abyssinica* and *N. reynaudiana*. A comparison of cpSSRs revealed a relative conservatism in repeat numbers and consistent with other reports (Wheeler et al., 2014; Jiang, Hinsinger & Strijk, 2016). Most of the cpSSR in *E. tef* is distributed in the noncoding region of the genome, which is consistent with other studies (Li et al., 2018; Abdullah et al., 2019). Chloroplast derived microsatellite markers were developed and utilized in various studies such as assessment of the maternal line of hybrid wheat (Tomar et al., 2014), genetic diversity and relationships analysis among potato accessions (Lee et al., 2019) and species differentiation (Decesare, Hodkinson & Barth, 2010). Our study provides cpSSRs data that could provide valuable molecular tools for the evolutionary studies of *E. tef*.

Although plastomes are highly conserved, there are hotspots region with SNPs and indels mutations, commonly used as DNA barcoding (Kress et al., 2005; Fan et al., 2018). These variations are uniparentally inherited and thus analytically attractive to trace the evolutionary history of maternal lines in the crop breeding program *(Keeling, 2010*; Tomar et al., 2014). In the present study, intraspecific chloroplast polymorphic sites were detected within the *E. tef* accessions. The 21 variable sites (12 InDels and 9 SNPs) identified in the present study include: *rps16 intron*, *trnM-CAU-trnE-UUC*, *atpE*, *petA-psbJ*, *clpP-psbB*, *psbB*, *ndhB, petB, psaC-ndhE, rpl16 intron, ccsA-ndhD, psaA-ycf3*, *trnT-UGU-trnS-UGA*, *ndhC-trnV-UAC*, *atpB-rbcL*, *psaJ-rpl33*, *rpl33-rps18*, *petD-rpoA*, *trnY-GAU-trnD-GUC*, *trnN-GUU-rps15.* The identified variable sites have provided valuable insight into the intraspecific genetic diversity in *E. tef* and could provide a valuable genomic resource for plastid marker development. The noncoding regions of plastomes have higher sequence variation than PCGs (Choi, Chung & Park, 2016; Skuza et al., 2019) and are widely used in population genetics and phylogenetic studies. This because in the genome, the PCGs is highly conserved than the noncoding regions (Cao et al., 2018; Wu et al., 2018). Similarly, in the current study, 81% of the identified SNPs and InDels markers were sited in the noncoding region of the plastid genomes. In general, nucleotide substitutions less frequently occur in PCGs than noncoding regions of plastomes (Kim et al., 2015; Daniell et al., 2016).

The nonsynonymous (ka) and synonymous (ks) substitution ratio (ka/ks) are widely used as an estimator for adaptive evolution on PCGs (Erixon & Oxelman, 2008; Gao et al., 2018). The fact that the positive selection in PCGs of plastomes viewed as an important driving force of adaptive evolution (Johnson & Melis, 2004; Zhong et al., 2009; Hu et al., 2015). We analyzed ka/ks ratio of four mutational PCGs of *E. tef* accessions, which indicated that all four mutational PCGs were under purifying selection (ka/ks < 1).

### Phylogenetic analysis

In previous studies, plastid markers have been used to determine the *E. tef* phylogenetic relationship (Espelund et al., 2000; Ingram, 2010). However, complete plastome and multi loci markers provide more detailed insight (Krawczyk et al., 2018; Wu et al., 2018). In this study, two datasets (complete plastome and twenty variable loci) were applied to determine whether the phylogenetic relationships of *E. tef* accessions reflected the biogeographic pattern. The phylogenetic tree has divided the accessions into two clusters with identical tree topologies. We found that phylogeny inferred from both datasets and analysis methods (BI and ML) have been able to delineate accessions from south Ethiopia (T3, T15, T24, T43, T51) with robust support (Fig. 3). Furthermore, patterns of mutations among accessions are consistent with all tree topologies. For example, several unique mutational sites were identified in accession from Eritrea (T16), which might be a reason for the relatively long branch length (Fig. 3). Overall, both datasets were able to provide the phylogenetic relationship with a more informative biogeographical pattern among the accessions from the south (Fig. 3) and also identify accession (T16) from Eritrea (Fig. 3). This indicated that the identified variable sites could be useful molecular markers in phylogenetic and biogeography studies. Phylogenetic relationships among *Eragrostis* have been investigated based on a small number of plastid loci *(rps16, trnL-UAA, trnL-trnF*) (Espelund et al., 2000; Ingram, 2010), but these have failed to provide intra-specific variations and sufficient phylogenetic signal of *E. tef*.

Despite the existence of clusters with a clear biogeographical pattern, the phylogenetic analysis did not reveal a robust biogeographical structure. For example, accessions from the western and central parts of the country are not clustered with their respective geographic origin. Similar analyses conducted in the previous study using the nuclear genome also did not show unambiguous geographic distribution patterns (Fikre, Tesfaye & Assefa, 2019). The lack of clear spatial structure may be attributed to gene flow between adjacent populations and seed exchange among farmers (Assefa, Merker & Tefera, 2004). We also infer that the limited geographical representation of our studied accessions might be the reason to contribute the insufficient geographical information.

## Conclusions

In this study, a comparison of 32 complete plastomes of *E. tef* accessions was performed and revealed a low level of sequence variability. Only 34 polymorphic sites (13 cpSSRs, 12 InDels and 9 SNPs) were identified in the plastome of these accessions. The noncoding regions of the genome exhibited higher variable sites than PCGs. The newly sequenced *E. tef* plastomes also provide an additional genomic resource for undertaking various studies in an economical crop.

Additionally, the phylogenetic tree provides an informative insight into the genetic relationship of sampled accessions with their biogeographic distribution. In the future, we would suggest expanded sampling of *E. tef* and its wild relatives need to be used for assessing the biogeography of this economically important crop. Genome-wide association study is also imperative to identify the genetic basis of agriculturally important traits in *E. tef*. Overall, in our study, the complete plastomes and detected variable sites could be a useful genomic resource for molecular breeding, identification, population genetics, and biogeography studies of *E. tef* and related crop species in the *Chloridoideae*.

##  Supplemental Information

10.7717/peerj.9314/supp-1 Supplemental Information 1 Comparisons of the LSC, SSC, IR region boundaries within 32 *E. tef* accessions

10.7717/peerj.9314/supp-2 Supplemental Information 2 Sequence identity plots based on 32 *E. tef* plastomes The vertical scale indicates the percentage of identity ranging from 50 to 100. Gene transcription direction was indicated by gray arrows.

10.7717/peerj.9314/supp-3 Supplemental Information 3 List of annotated genes in *E. tef* accessions

10.7717/peerj.9314/supp-4 Supplemental Information 4 List of cpSSRs in the plastome of E. tef species

10.7717/peerj.9314/supp-5 Supplemental Information 5 List of cpSSRs markers with primer sequence and genome position in the *E. tef.*

10.7717/peerj.9314/supp-6 Supplemental Information 6 CpSSR types and amount in the *E. tef, E. minor, N. reynaudiana* and *M. abyssinica* plastomes

10.7717/peerj.9314/supp-7 Supplemental Information 7 The distribution of long repeat sequences identified in *E. tef* plastomes by REPuter software

10.7717/peerj.9314/supp-8 Supplemental Information 8 Primer pairs designed for the identified SNPs and InDels in the plastomes of *E. tef* The distribution of long repeat sequence identified in *E. tef* plastomes by REPuter.

10.7717/peerj.9314/supp-9 Supplemental Information 9 Annotated sequence data T3

10.7717/peerj.9314/supp-10 Supplemental Information 10 Annotated sequence data T4

10.7717/peerj.9314/supp-11 Supplemental Information 11 Annotated sequence T10

10.7717/peerj.9314/supp-12 Supplemental Information 12 Annotated sequence data T11

10.7717/peerj.9314/supp-13 Supplemental Information 13 Annotated sequence data T12

10.7717/peerj.9314/supp-14 Supplemental Information 14 Annotated sequence T14

10.7717/peerj.9314/supp-15 Supplemental Information 15 Annotated sequence data T15

10.7717/peerj.9314/supp-16 Supplemental Information 16 Annotated sequence data T16

10.7717/peerj.9314/supp-17 Supplemental Information 17 Annotated sequence data T20

10.7717/peerj.9314/supp-18 Supplemental Information 18 Annotated sequence data T21

10.7717/peerj.9314/supp-19 Supplemental Information 19 Annotated sequence data T24

10.7717/peerj.9314/supp-20 Supplemental Information 20 Annotated sequence data T34

10.7717/peerj.9314/supp-21 Supplemental Information 21 Annotated sequence data T36

10.7717/peerj.9314/supp-22 Supplemental Information 22 Annotated sequence data T43

10.7717/peerj.9314/supp-23 Supplemental Information 23 Annotated sequence data T44

10.7717/peerj.9314/supp-24 Supplemental Information 24 Annotated sequence data T45

10.7717/peerj.9314/supp-25 Supplemental Information 25 Annotated sequence data T49

10.7717/peerj.9314/supp-26 Supplemental Information 26 Annotated sequence data T50

10.7717/peerj.9314/supp-27 Supplemental Information 27 Annotated sequence data T51

10.7717/peerj.9314/supp-28 Supplemental Information 28 Annotated sequence data T56

10.7717/peerj.9314/supp-29 Supplemental Information 29 Annotated sequence data 66

10.7717/peerj.9314/supp-30 Supplemental Information 30 Annotated sequence data 68

10.7717/peerj.9314/supp-31 Supplemental Information 31 Annotated sequence data 69

10.7717/peerj.9314/supp-32 Supplemental Information 32 Annotated sequence data 74

10.7717/peerj.9314/supp-33 Supplemental Information 33 Annotated sequence data T90

10.7717/peerj.9314/supp-34 Supplemental Information 34 Annotated sequence data T1-2

10.7717/peerj.9314/supp-35 Supplemental Information 35 Annotated sequence data T81

10.7717/peerj.9314/supp-36 Supplemental Information 36 Annotated sequence data 93

10.7717/peerj.9314/supp-37 Supplemental Information 37 Annotatee sequence data T100

10.7717/peerj.9314/supp-38 Supplemental Information 38 Annotated sequence data T101

10.7717/peerj.9314/supp-39 Supplemental Information 39 Annotated sequence data T115

10.7717/peerj.9314/supp-40 Supplemental Information 40 Annotated sequence data T116

10.7717/peerj.9314/supp-41 Supplemental Information 41 Thirty two (32) plastome sequence data

## Additional Information and Declarations

### Field Study Permissions

The following information was supplied relating to field study approvals (i.e., approving body and any reference numbers): Ethiopian Biodiversity Institute (EBI) approved this study (EBI 71 22 2294 2018).

### Data Availability

The following information was supplied regarding data availability: Data is available at the National Center for Biotechnology Information (NCBI): MN780987 to MN781018. Raw data are available in the Supplemental Files.
