# Supplementary figures and images for: Comparative analyses of 32 complete plastomes of Tef (Eragrostis tef ) accessions from Ethiopia: phylogenetic relationships and mutational hotspots
Source: PeerJ. 2020 Jun 19;8:e9314. doi: 10.7717/peerj.9314 (PMC7307559; doi:10.7717/peerj.9314)

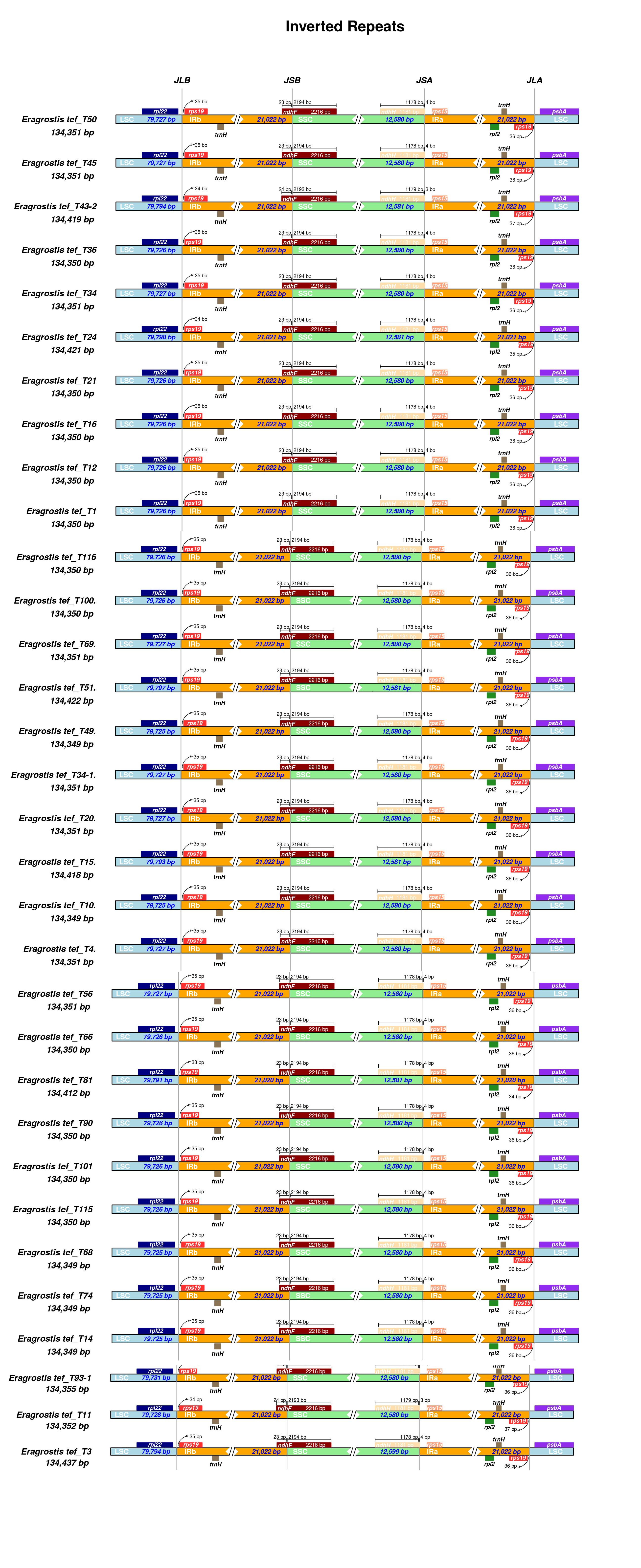

Supplement: Supplemental Information 1 [file peerj-08-9314-s001.png]

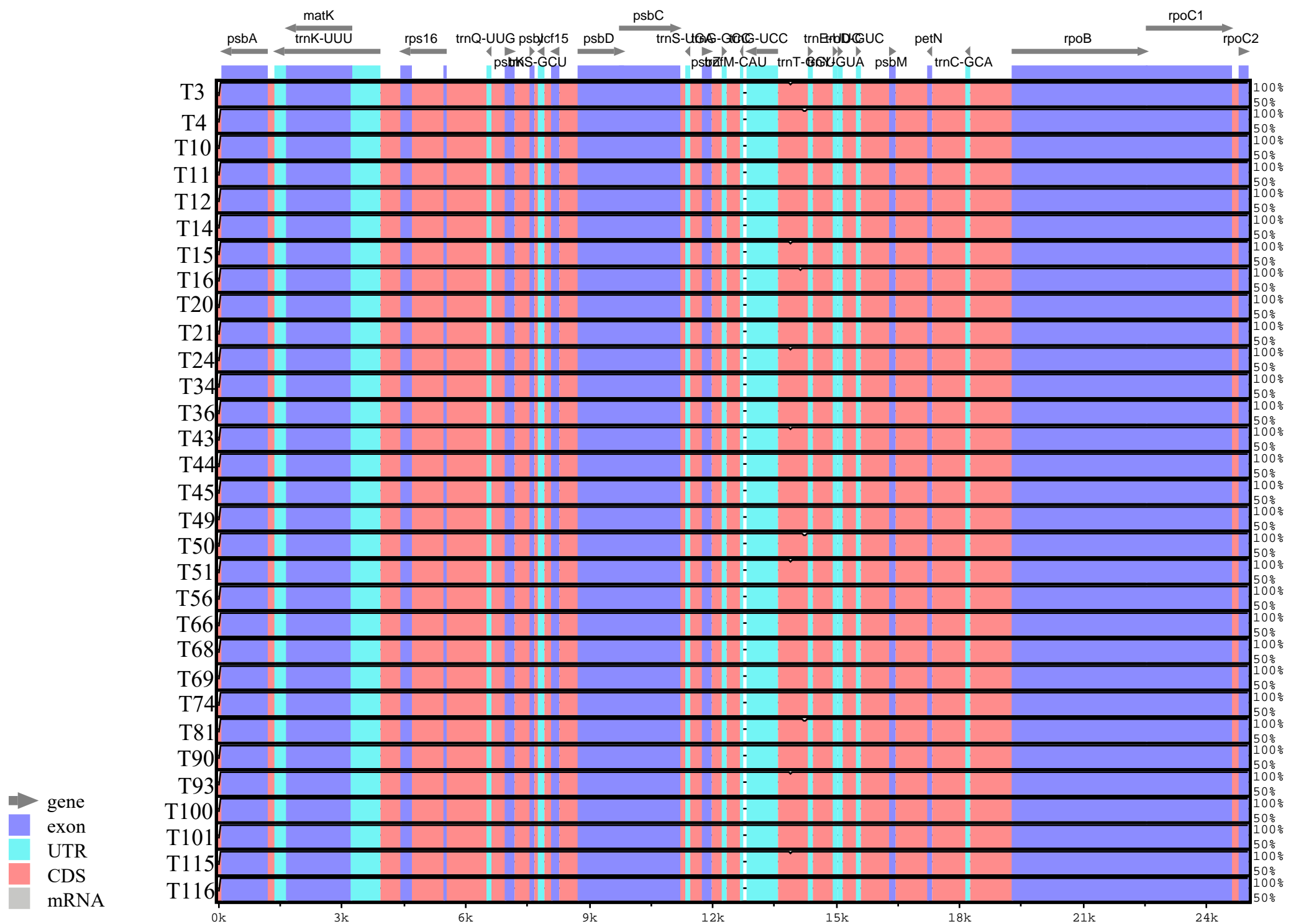

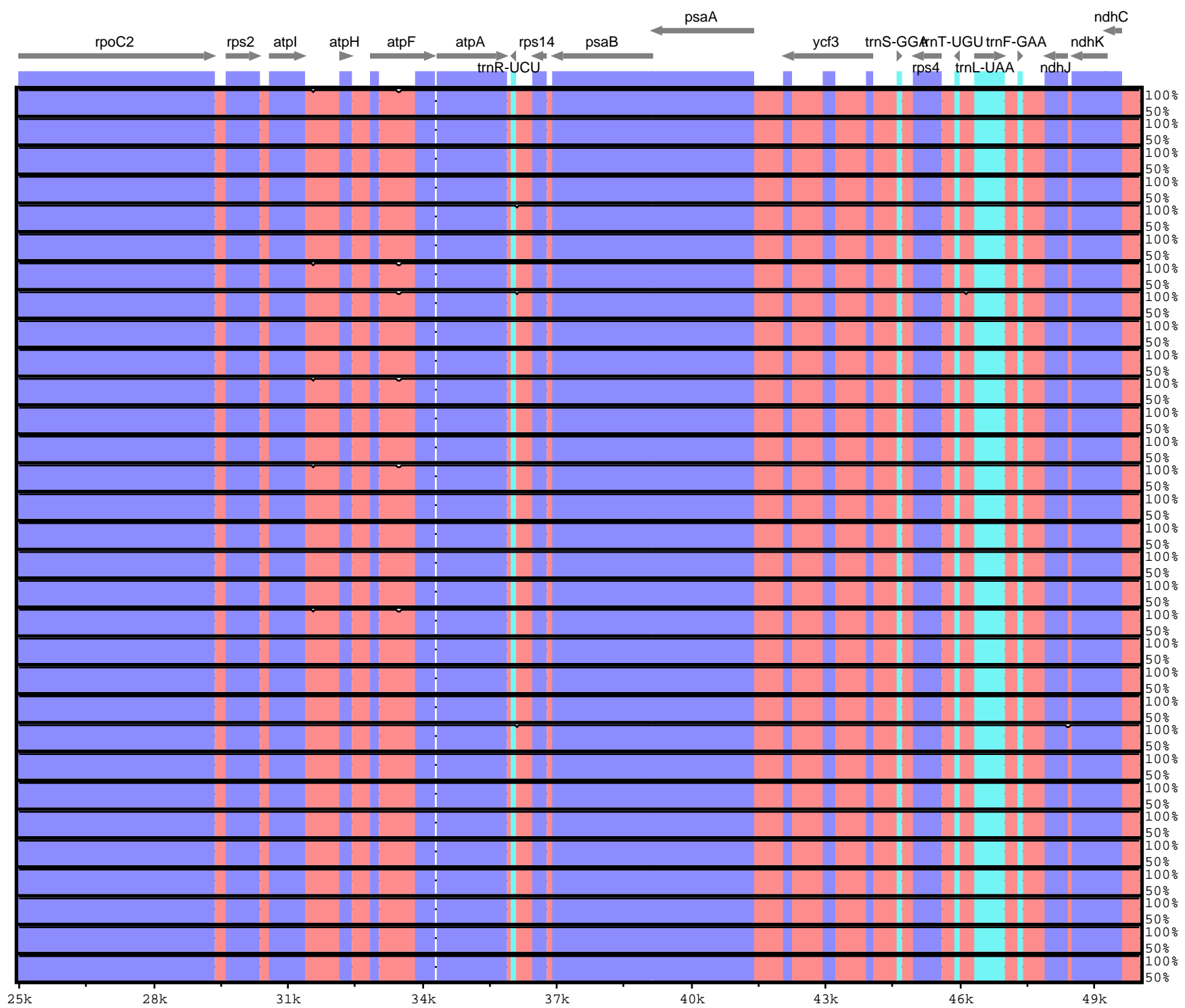

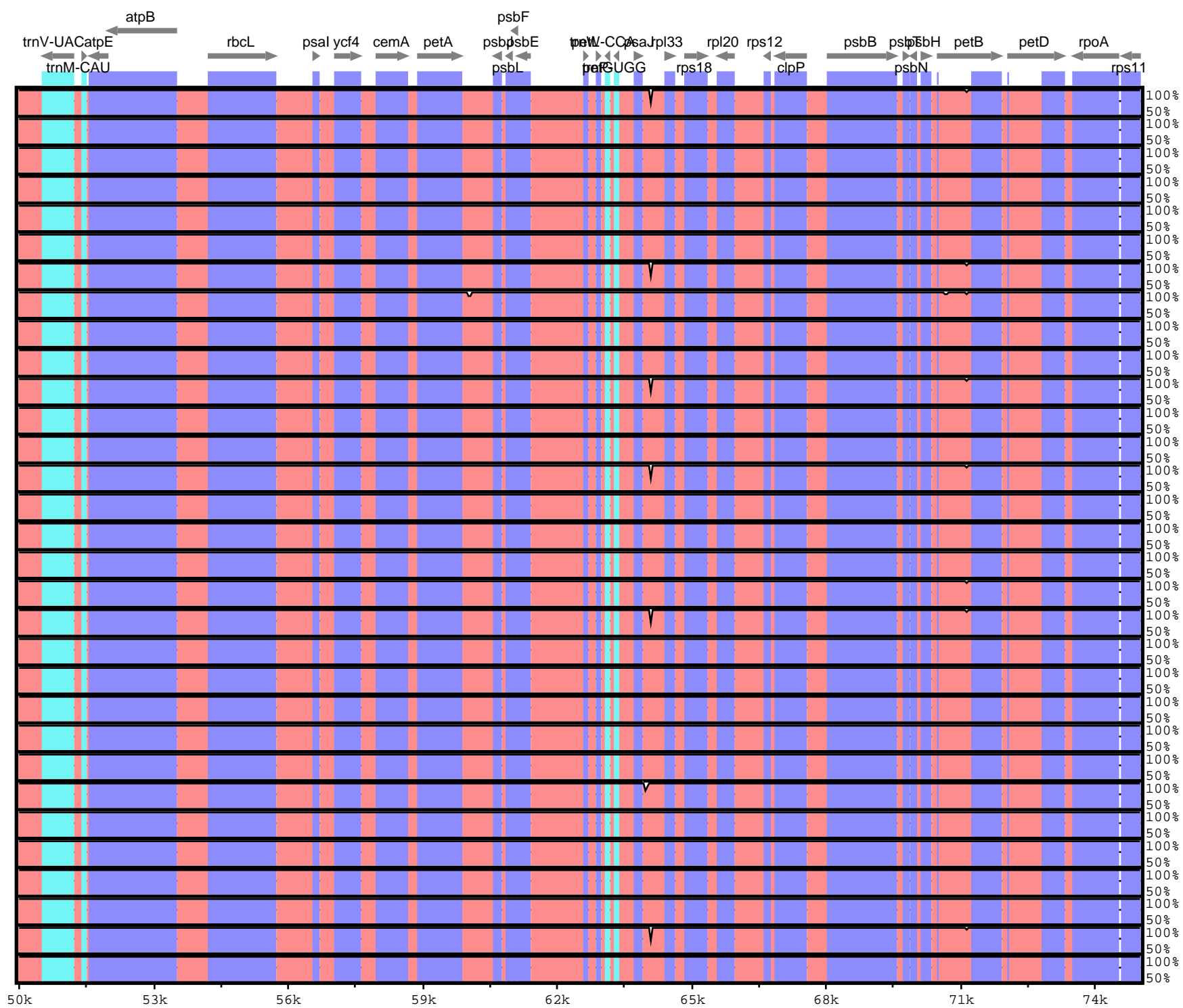

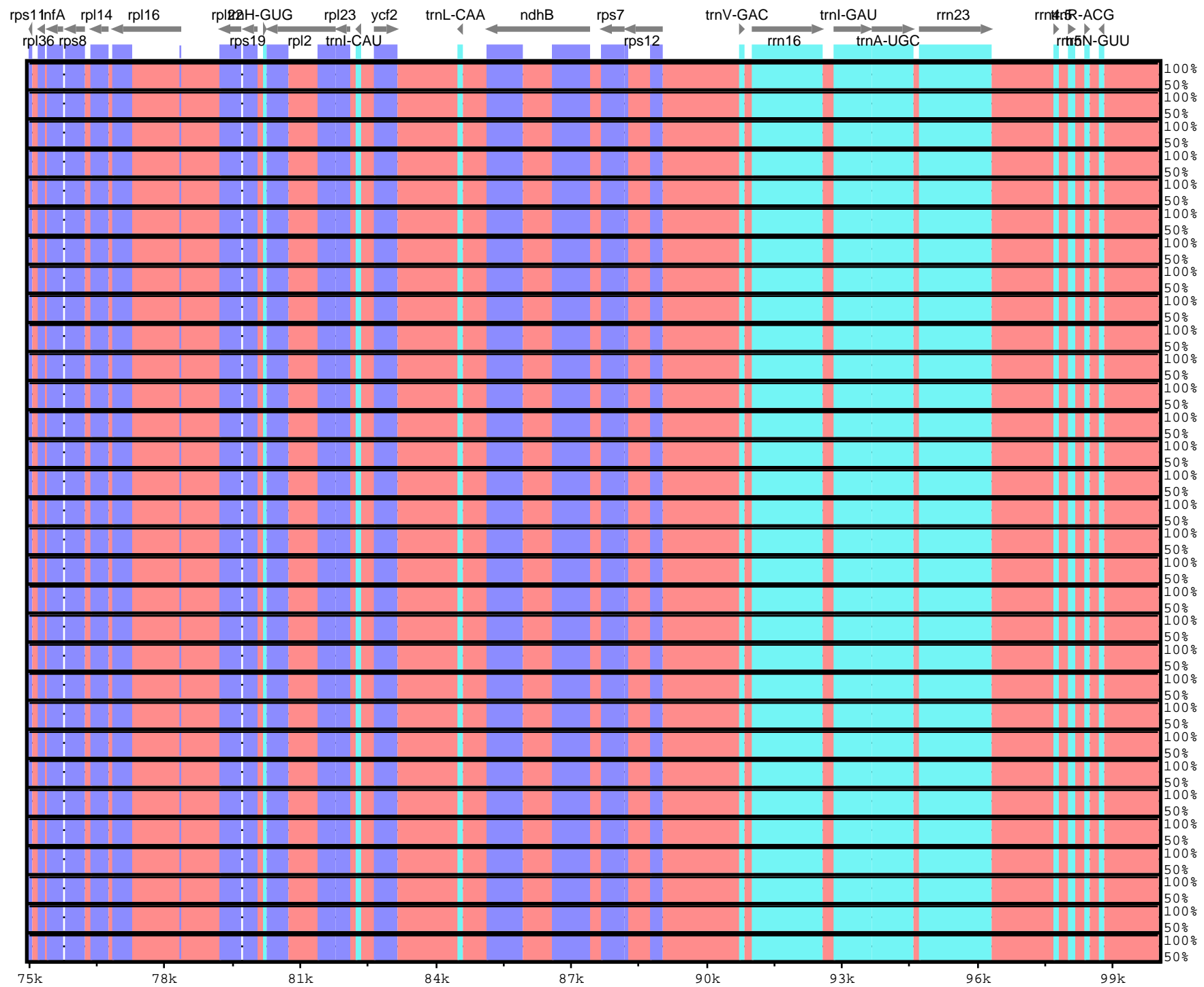

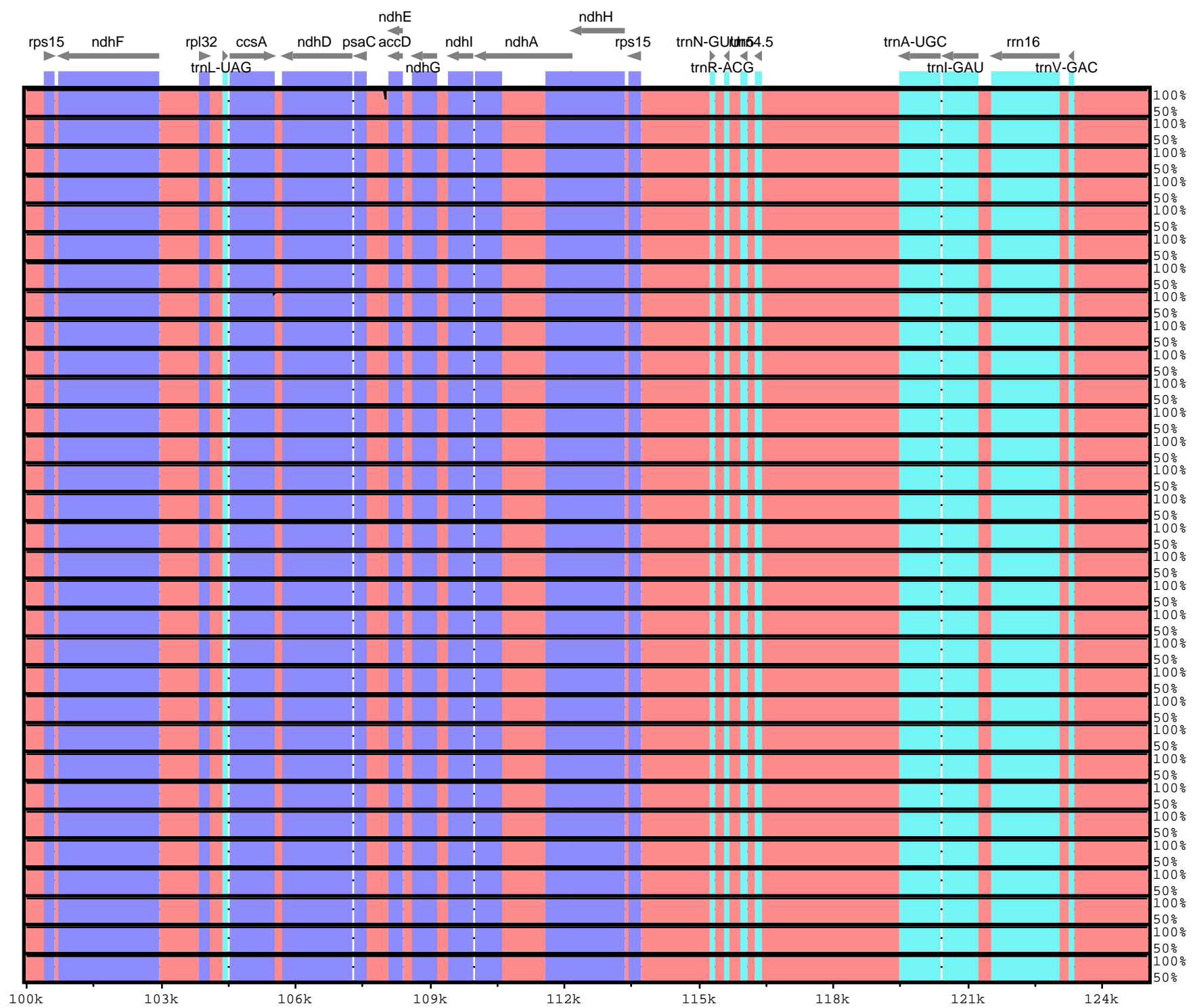

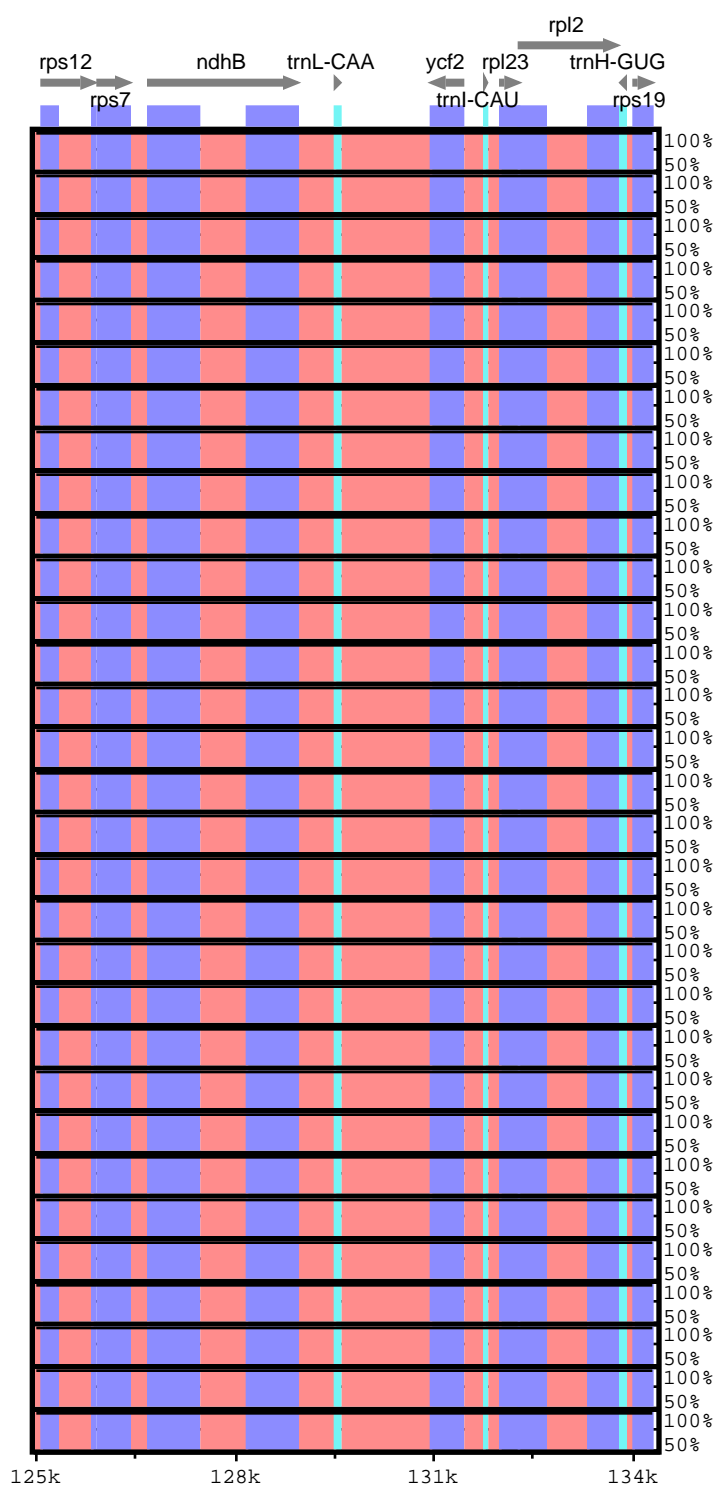

Supplement: Supplemental Information 2 — The vertical scale indicates the percentage of identity ranging from 50 to 100. Gene transcription direction was indicated by gray arrows. [file peerj-08-9314-s002.pdf]
