# Supplementary material for: Comparative analyses of 32 complete plastomes of Tef (Eragrostis tef ) accessions from Ethiopia: phylogenetic relationships and mutational hotspots
Source: PeerJ. 2020 Jun 19;8:e9314. doi: 10.7717/peerj.9314 (PMC7307559; doi:10.7717/peerj.9314)
Supplement: Supplemental Information 3 [file peerj-08-9314-s003.docx]

**Table S1** List of annotated genes in *E. tef* accessions

| group of genes | genes names |
| --- | --- |
| Photosystem I | *psaA, psaB, psaC, psaL, psaJ* |
| Photosystem II | *psbA, psbB, psbC, psbD, psbE, psbF, psbH, psbI, psbJ, psbK, psbL, psbM, psbN, psbT, psbZ* |
| Cytochrome b/f complex | *petA, petB*, petD*, petG, petL, petN* |
| ATP synthase | *atpA, atpB, atpE, atpF*, atpH, atpI* |
| NADH dehydrogenase | *ndhA*********, ndhB******** *(×2), ndhC, ndhD, ndhE, ndhF, ndhG, ndhH, ndhI, ndhJ, ndhK* |
| RubisCO large subunit | *rbcL* |
| RNA polymerase | *rpoA, rpoB, rpoC1, rpoC2* |
| Ribosomal proteins (SSC) | *rps2, rps3, rps4, rps7 (×2), rps8, rps11, rps12*********(×2), rps14, rps15(×2), rps16*********, rps18, rps19(×2)* |
| Ribosomal proteins (LSC) | *rpl2*(×2) , rpl14, rpl16*, rpl20, rpl22, rpl23, rpl32, rpl33, rpl36* |
| protease proteolytic subunit and maturase K | *clpP, matK* |
| other gene | *infA, ccsA, cemA* |
| hypothetical chloroplast reading frames (ycf) | *ycf68 (×2), ycf3*, ycf4,* |
| Ribosomal RNAs | *rrn4.5 (×2), rrn5 (×2), rrn16 (×2), rrn23 (×2)* |
| transfer RNAs | *trnH-GUG(×2), trnI-CAU(×2), trnL-CAA(×2), trnV-GAC(×2), trnI-GAU*(×2), trnA-UGC*(×2), trnR-ACG(×2), trnN-GUU(×2), trnP-UGG, trnW-CCA, trnV-UAC*, trnT-UGU, trnfM-CAU, trnR-UCU, trnC-GCA, trnG-UCC*, trnfM-CAU, trnS-UGA, trnS-GCU, trnQ-UUG, trnK-UUU*, trnM-CAU, trnF-GAA, trnL-UAA, trnS-GGA, trnD-GUC, trnY-GUA ,trnE-UUC, trnM-CAU, trnG-GCC, trnL-UAG* |

(×2) Two gene copies in the IRs, gene containing introns (*)
