# Supplementary material for: Comparative analyses of 32 complete plastomes of Tef (Eragrostis tef ) accessions from Ethiopia: phylogenetic relationships and mutational hotspots
Source: PeerJ. 2020 Jun 19;8:e9314. doi: 10.7717/peerj.9314 (PMC7307559; doi:10.7717/peerj.9314)
Supplement: Supplemental Information 5 [file peerj-08-9314-s005.docx]

**Table S3** List of cpSSRs markers with primer sequence and genome position in the *E. tef*

| cpSSR  markers | Position | Reverse primer | Forward primer | Region | TM* | Size (bp) |
| --- | --- | --- | --- | --- | --- | --- |
| *tefcpSSR1* | *matK-rps16* | TGAAGCAACAAATTCGTCCA | GATCGGGGAATCCTTTCAAT | LSC | 60 | 238 |
| *tefcpSSR2* | *rps16-trnQ-UUG* | TCTGTCAGGCACATTCAGGA | CGAATCCTTCCGTCCCAGAT | LSC | 59 | 237 |
| *tefcpSSR3* | *trnS-GCU-psbD* | CCCTCTTTTCTCATTCGCAG | AGACTTAGACCGCGCAAGAA | LSC | 59 | 212 |
| *tefcpSSR4* | *psbZ-trnG-GCC* | CTATGTCTGGGTCGACCGTT | AAAATGCTATTTGCGTTCCG | LSC | 60 | 137 |
| *tefcpSSR5* | *trnT-GGU-trnM-CAU* | TCAAAAATTCGCCACTTTCA | TTCTCATCATAAGATCAGCCAA | LSC | 59 | 252 |
| *tefcpSSR6* | *trnC-GCA-rpoB* | ATGGCAAAGGAGCAAAAATG | ACATAAGGGGGAGCCCTAGA | LSC | 59 | 230 |
| *tefcpSSR7* | *psbM-petN* | GCATCCACTTAATTTCAAGCAA | GAAGCATCTCGCGTCATTTT | LSC | 60 | 176 |
| *tefcpSSR8* | *rpoC2-rps2* | TGGCCAATTCTGAGTAGAAGG | GGGCCATTTTAGGATTCCAT | LSC | 59 | 238 |
| *tefcpSSR9* | *atpI-atpH* | TCCATGGAAGGTCATCATTG | CAAAATCATTCCGCACAAAA | LSC | 59 | 278 |
| *tefcpSSR10* | *atpF intron* | TCATGGTCAGCAAAGTTGTTTC | CCTTTTTCCAATGCCGAAT | LSC | 60 | 186 |
| *tefcpSSR11* | *trnR-UCU-trnfM-CAU* | CCATTAGACAATGGACGCTTT | ATCCACTAGTTCCCCGCTCT | LSC | 60 | 275 |
| *tefcpSSR12* | *ndhJ-ndhK* | ACCGGAACTTACGAGCAAGA | TAGATAACCACCCCTGCTGC | LSC | 59 | 243 |
| *tefcpSSR13* | *rpl16 intron* | GCGAATGAAATGAGAAAGCG | GCGAATGAAATGAGAAAGCG | LSC | 59 | 279 |

Note: cpSSR: chloroplast simple sequence repeat;*TM: Temperature
