# Supplementary material for: Comparative analyses of 32 complete plastomes of Tef (Eragrostis tef ) accessions from Ethiopia: phylogenetic relationships and mutational hotspots
Source: PeerJ. 2020 Jun 19;8:e9314. doi: 10.7717/peerj.9314 (PMC7307559; doi:10.7717/peerj.9314)
Supplement: Supplemental Information 6 [file peerj-08-9314-s006.docx]

**Table S4** CpSSR types and amount in the *E. tef, E. minor, N. reynaudiana and M. abyssinica* plastomes

| SSR type | Repeat unit | *E. tef* | *E. minor* | *N. reynaudiana* | *M. abyssinica* |
| --- | --- | --- | --- | --- | --- |
| Mono- | A/T | 130 | 124 | 126 | 107 |
|  | C/G | 5 | 5 | 3 | 5 |
| Di- | AG/CT | 1 | 1 | 1 | 1 |
|  | AT/AT | 5 | 7 | 7 | 3 |
| Tri- | AAG/CTT | 1 | 1 | 1 | 1 |
|  | AAT/ATT | 0 | 2 | 0 | 1 |
| Tetra- | ACCT/AGGT | 1 | 1 | 1 | 0 |
| Penta- | AAAAT/ATTTT | 0 | 0 | 1 | 0 |
|  | AATAG/ATTCT | 0 | 0 | 2 | 0 |
| Total |  | 143 | 141 | 142 | 118 |
