# Supplementary material for: Comparative analyses of 32 complete plastomes of Tef (Eragrostis tef ) accessions from Ethiopia: phylogenetic relationships and mutational hotspots
Source: PeerJ. 2020 Jun 19;8:e9314. doi: 10.7717/peerj.9314 (PMC7307559; doi:10.7717/peerj.9314)
Supplement: Supplemental Information 7 [file peerj-08-9314-s007.docx]

**Table S5** The distribution of long repeat sequences identified in *E. tef* plastomes by REPuter software

| Repeat 1 (start position) | Location 1 | Repeat 2 (start location) | Location | Size | Region | Type |
| --- | --- | --- | --- | --- | --- | --- |
| 55995 | *rbcL-psaI* | 81905 | *rpl23* | 237 | LSC | P |
| 55995 | *rbcl-psaI* | 132089 | *trnM-CAU* | 237 | LSC | F |
| 56059 | *rbcl-psaI* | 81905 | *rpl23* | 173 | LSC | P |
| 56059 | *rbcl-psaI* | 132153 | *rpl23* | 173 | LSC | F |
| 63973 | *trnP-UGG-psaJ* | 64033 | *psaJ-rpl23* | 120 | LSC | F |
| 56148 | *rbcl-psaI* | 81905 | *rpl23* | 84 | LSC | P |
| 56148 | *rbcl-psaI* | 132242 | *rpl23* | 84 | LSC | F |
| 63973 | *psaJ-rpl33* | 64093 | *psaJ-rpl23* | 60 | LSC | F |
| 100209 | *trnN-GUU-rps15* | 100209 | *trnN-GUU-rps15* | 52 | IRb | P |
| 100209 | *trnN-GUU-rps15* | 113970 | *rps15-trnN-GUU* | 52 | IRb | F |
| 113970 | *rps15-trnN-GUU* | 113970 | *rps15-trnN-GUU* | 52 | IRa | P |
| 64949 | *rps18* | 65012 | *rps18* | 52 | LSC | F |
| 69917 | *psbT-psbN* | 69917 | *psbT-psbN* | 48 | LSC | P |
| 12781 | *trnM-CAU-trnG-UCC* | 36365 | *trnM-CAU-trnG-UCC* | 46 | LSC | F |
| 13751 | *trnG-UCC-trnT-GGU* | 81565 | *rpl2* | 45 | LSC | F |
| 13751 | *trnG-UCC-trnT-GGU* | 132621 | *rpl2* | 45 | LSC | P |
| 64967 | *rps18* | 65009 | *rps18* | 44 | LSC | F |
| 64970 | *rps18* | 65033 | *rps18* | 31 | LSC | F |
| 26765 | *rpoC2* | 26999 | *rpoC2* | 37 | LSC | F |
| 16421 | *psbM* | 16421 | *PsbM* | 30 | LSC | P |
| 7783 | *psbI-trnS-GCU* | 44668 | *trnS -GGA* | 36 | LSC | P |
| 12791 | *trnM-CAU-trnG-UCC* | 36375 | *trnM-CAU-rps14* | 36 | LSC | F |
| 43231 | *ycf3* | 89120 | *rps12-trnV-GAC* | 36 | LSC | F |
| 43231 | *ycf3* | 125075 | *trnV-GAC-rps12* | 36 | LSC | P |
| 65008 | *rps18* | 65029 | *rps18* | 38 | LSC | F |
| 26770 | *rpoC2* | 27082 | *rpoC2* | 37 | LSC | F |
| 64946 | *rps18* | 65030 | *rps18* | 37 | LSC | F |
| 26824 | *rpoC2* | 26866 | *rpoC2* | 34 | LSC | F |
| 1752 | *matK* | 77960 | *rpl16* | 30 | LSC | F |
| 27004 | *rpoC2* | 27082 | *rpoC2* | 32 | LSC | F |
| 26759 | *rpoC2* | 26918 | *rpoC2* | 34 | LSC | F |
| 26924 | *rpoC2* | 26999 | *rpoC2* | 34 | LSC | F |
| 38291 | *psaB* | 40515 | *PsaA* | 34 | LSC | F |
| 31948 | *atpI-atpH* | 31948 | *atpI-atpH* | 30 | LSC | R |
| 65395 | *rps18* | 65395 | *rps18* | 30 | LSC | P |
| 86124 | *ndhB (intron)* | 86124 | *ndhB (intron)* | 30 | IRb | P |
| 86124 | *ndhB (intron)* | 128077 | *ndhB (intron)* | 30 | IRb | F |
| 128077 | *ndhB (intron)* | 128077 | *ndhB (intron)* | 30 | IRa | P |
| 11330 | *psbC-trnS-UGA* | 44675 | *trnS-GGA* | 32 | LSC | P |
| 14398 | *trnM-CAU* | 45892 | *rps4-trnT-UGU* | 31 | LSC | P |
| 26810 | *rpoC2* | 26852 | *rpoC2* | 31 | LSC | F |
| 64949 | *rps18* | 64970 | *rps18* | 31 | LSC | F |
| 64995 | *rps18* | 65037 | *rps18* | 30 | LSC | F |
| 100236 | *trnN-GUU-rps15* | 113997 | *rps15-trnN-GUU* | 30 | IRb | F |
