# Supplementary material for: Comparative analyses of 32 complete plastomes of Tef (Eragrostis tef ) accessions from Ethiopia: phylogenetic relationships and mutational hotspots
Source: PeerJ. 2020 Jun 19;8:e9314. doi: 10.7717/peerj.9314 (PMC7307559; doi:10.7717/peerj.9314)
Supplement: Supplemental Information 8 — The distribution of long repeat sequence identified in E. tef plastomes by REPuter. [file peerj-08-9314-s008.docx]

**Table S6** Primer pairs designed for the identified SNPs and InDels in the plastomes of *E. tef*

| Markers name | Position | Reverse primer | Forward primer | Region | TM* | Size (bp) |
| --- | --- | --- | --- | --- | --- | --- |
| InDels markers | | | | | | |
| *E.tef 1* | *trnY-GAU-trnD-GUC* | AGCTCGGCCCAAGAATCTAG | GCCTTCTGCCCCTTTTATTCA | LSC | 59 | 515 |
| *E.tef 2* | *psaA-ycf3* | TACCTCTCTGTAGCACCCGT | CGGGTGTATTGTGGCGATTC | LSC | 59 | 684 |
| *E.tef 3* | *trnT-UGU-trnS-UGA* | GGATTTGAACCGATGACCCTC | ACAATCAAGTCCGTAGCGTC | LSC | 58 | 504 |
| *E.tef 4* | *ndhC-trnV-UAC* | TGGAGAAAATCGTTGCAGTCA | GAGCACAGGGAGCCATCTTA | LSC | 58 | 502 |
| *E.tef 5* | *atpB-rbcL* | TGCCTACTCTACTTTCCTGTCT | TACTCGGAATGCTGCCAAGA | LSC | 58 | 634 |
| *E.tef 6* | *psaJ-rpl33* | TACCTCTCTGTAGCACCCGT | CGGGTGTATTGTGGCGATTC | LSC | 59 | 684 |
| *E.tef 7* | *rpl33-rps18* | TACCTCTCTGTAGCACCCGT | CTTGCCCATGAACCTCCTTT | LSC | 59 | 623 |
| *E.tef 8* | *petD-rpoA* | AAATCCATTTCGTCGCCCAG | TACACGCAGAGGAAGAAGGC | LSC | 58 | 667 |
| *E.tef 9* | *trnN-GUU-rps15* | TTCTTCGTAGTGGCTCCGTT | ACCTGTGAAGATCGTGCATT | IR | 59 | 580 |
| *E.tef 10* | *ccsA-ndhD* | TCGTGTCATTAGTCTCGGGT | CGCTATCCGTTGACAGGGTA | LSC | 58 | 541 |
| *E.tef 11* | *petA-psbJ* | CTCCCGGACCAGAACTTCTT | GGACCCGCGGAATTCCTATA | LSC | 59 | 900 |
| *E.tef 12* | *psaC-ndhE* | TGTAGACACCAGACGAAGCA | TCACAAGCCGAAACATGGTT | SSC | 58 | 677 |
| SNP markers | | | | | | |
| *E.tef 1* | *rps16 intron* | GCTCCTCGCGAATGAAATGA | TAAAGAGCAGCCCGGGTTAA | LSC | 58 | 517 |
| *E.tef 2* | *trnM-CAU-trnE-UUC* | AGCTTAGCCCAATTCATGCA | TCCAACCAACCAACCTTTCA | LSC | 57 | 573 |
| *E.tef 3* | *atpE* | ATTGGCGCACGTGTAAGC | TTCTGTGGAGTGGTTTTGCG | LSC | 59 | 648 |
| *E.tef 4* | *clpP-psbB* | GCGTGAGGGAATGCTATACG | ACACGATACCAAGGCAAACC | LSC | 58 | 895 |
| *E.tef 5* | *psbB* | TGTGAGAGCAGACGTTCCTT | TTAGTCGGAACCTTAGGCGG | LSC | 59 | 657 |
| *E.tef6* | *petB* | GGAAAGTAGCTCCGGGTTG | ACCAAAAGGCAAGGGTTCTTC | LSC | 57 | 627 |
| *E.tef 7* | *rpl16 intron* | GCTCCTCGCGAATGAAATGA | TAAAGAGCAGCCCGGGTTAA | LSC | 58 | 517 |
| *E.tef 8* | *psaC-ndhE* | TGTAGACACCAGACGAAGCA | TCACAAGCCGAAACATGGTT | SSC | 58 | 677 |
| *E.tef 9* | *ndhB* | CTTGCCATCCACACCAGAAT | CCTACGGAACCAAGGTCGAA | IR | 58 | 774 |

*TM Temperature
